# Bone regeneration of minipig mandibular defect by adipose derived mesenchymal stem cells seeded tri-calcium phosphate- poly(D,L-lactide-co-glycolide) scaffolds

**DOI:** 10.1038/s41598-020-59038-8

**Published:** 2020-02-06

**Authors:** Florian Andreas Probst, Riham Fliefel, Egon Burian, Monika Probst, Matthias Eddicks, Matthias Cornelsen, Christina Riedl, Hermann Seitz, Attila Aszódi, Matthias Schieker, Sven Otto

**Affiliations:** 1 Department of Oral and Maxillofacial Surgery and Facial Plastic Surgery, University Hospital, Ludwig-Maximilians-University, Munich, 80337 Germany; 2 Laboratory of Experimental Surgery and Regenerative Medicine (ExperiMed), Clinic for General, Trauma and Reconstructive Surgery, Ludwig-Maximilians-University, Munich, 80336 Germany; 3 Department of Oral and Maxillofacial Surgery, Faculty of Dentistry, Alexandria University, Alexandria, 21514 Egypt; 4 Department of Neuroradiology, Klinikum rechts der Isar, Technical University Munich, Munich, 81675 Germany; 5 Clinic for Swine, Center for Clinical Veterinary Medicine, Ludwig-Maximilians-University, Oberschleissheim, 85764 Germany; 6 Fluid Technology and Microfluidics, University of Rostock, Rostock, 18059 Germany

**Keywords:** Orthopaedics, Experimental models of disease, Preclinical research, Stem-cell research, Translational research

## Abstract

Reconstruction of bone defects represents a serious issue for orthopaedic and maxillofacial surgeons, especially in extensive bone loss. Adipose-derived mesenchymal stem cells (ADSCs) with tri-calcium phosphates (TCP) are widely used for bone regeneration facilitating the formation of bone extracellular matrix to promote reparative osteogenesis. The present study assessed the potential of cell-scaffold constructs for the regeneration of extensive mandibular bone defects in a minipig model. Sixteen skeletally mature miniature pigs were divided into two groups: Control group and scaffolds seeded with osteogenic differentiated pADSCs (n = 8/group). TCP-PLGA scaffolds with or without cells were integrated in the mandibular critical size defects and fixed by titanium osteosynthesis plates. After 12 weeks, ADSCs seeded scaffolds (*n* = 7) demonstrated significantly higher bone volume (34.8% ± 4.80%) than scaffolds implanted without cells (*n* = 6, 22.4% ± 9.85%) in the micro-CT (*p* < 0.05). Moreover, an increased amount of osteocalcin deposition was found in the test group in comparison to the control group (27.98 ± 2.81% vs 17.10 ± 3.57%, p < 0.001). In conclusion, ADSCs seeding on ceramic/polymer scaffolds improves bone regeneration in large mandibular defects. However, further improvement with regard to the osteogenic capacity is necessary to transfer this concept into clinical use.

## Introduction

Maxillofacial bone defects, which occur due to trauma, craniofacial deformities, tumour or infection can lead to facial deformities and severe maxillofacial dysfunctions provoking a dramatic decrease in the quality of life of the patients^1,2^.

The reconstruction of large bone defects poses many challenges in oral and maxillofacial surgery. Although autologous bone grafts are considered the gold standard in bone defect repair, there are some concerns related to the limited supply and donor site morbidity^3^.

Several alternatives as allografts, xenografts or synthetic bone substitutes have been brought by researchers and clinicians to restore the function and architecture of the defective bone but still cannot solve the problem due to various limitations. The search of new treatment alternatives has emerged enormously in the past few years. Among these alternatives, bone tissue-engineering (BTE) have been described as a promising technology to reconstruct bone defects in oral and maxillofacial surgery and increase clinical options for regeneration of maxillofacial osseous tissues^2,4,5^.

The regeneration of the defective bone with the application of BTE promotes 3D tissue model constructions by combining cells and/or inductive morphogenetic signals onto three-dimensional (3D) porous scaffolds in order to restore normal organ function^6,7^.

The use of stem cells (SCs) had been the base point in bone tissue engineering. Various SCs were used as embryonic SCs, adult somatic SCs and induced pluripotent SCs. Adult stem cells derived from various tissues such as skin, adipose tissue, bone marrow, and umbilical cord have been isolated and subjected to cell proliferation and differentiation assays leading to tissue regeneration^6,8^.

Besides to the use of bone marrow derived mesenchymal stem cells (BMSCs), adipose-derived mesenchymal stem cells (ADSCs) have recently become the focus in BTE due to their increased proliferation properties, their promising osteogenic capabilities and minimally invasive harvesting procedures^9,10^.

Innovations in BTE have led to the development of new biomaterials that resemble the 3D bone structure, in terms of mechanical properties as well as osteoconductive, osteoinductive, and osteogenic features^11^. Calcium phosphate ceramics (CPCs) have been widely used in bone tissue engineering as well as in orthopaedics and oral and maxillofacial surgery. Among the CPCs, tri-calcium phosphate (TCP) bioceramics are quite distinct for hard tissue regeneration due to their biocompatibility, degradation and new bone formation. Recently, composites of the bioceramic scaffolds with biodegradable polymers like poly (lactic-co-glycolic) acid (PLGA) were developed with the aim of increasing mechanical stability and improving tissue interaction^12–14^.

Cell-scaffold interactions have shown to significantly improve stem cell viability and guiding stem cell differentiation facilitating the formation of bone extracellular matrix to promote reparative osteogenesis^12,15^.

In order to identify whether bone tissue engineering might be a real alternative to the autologous bone grafts being the clinical gold standard, the present study established procedures for isolation and culture of pig ADSCs, to assess their cell viability and differentiation capacity into the osteogenic lineage in 2D and 3D cell culture; and finally to assess the potential of cell-scaffold assembly (TCP-PLGA scaffolds seeded with ADSCs) for the regeneration of extensive mandibular bone defects in a miniature pig model.

## Results

### Scaffold evaluation

**Figure 1:**
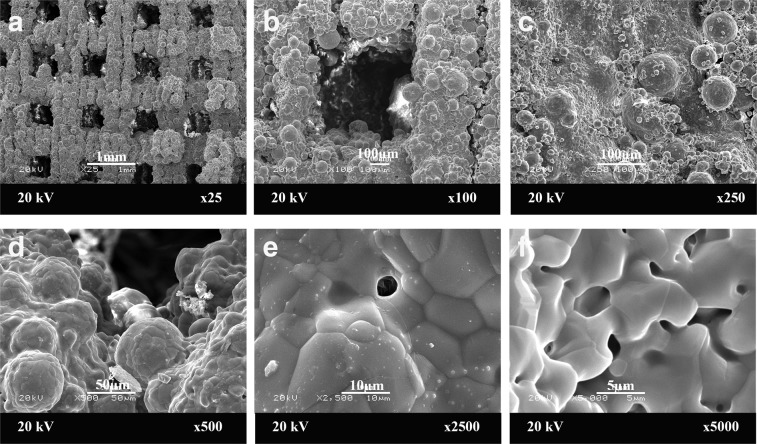
Scaffold characterization. The morphology of tri-calcium phophate poly(D,L-lactic-co-glycolic) acid (TCP-PLGA) scaffold observed by scanning electron microscopy (SEM) at low (**a–c**) and high magnifications (**d–f**) with interconnecting channels measuring about 450–500 μm. Scale bars represent 1 mm (**a**), 100 μm (**b**,**c**) and 50 μm (**d**), 10 µm (**e**) and 5 µm (**f**).

The average volume of the scaffolds was 7.15 cm³ (SD ± 1.10 cm³, range 5.58–8.80 cm³). The structure of the scaffold evaluated by scanning electron microscope (SEM) showed that tri-calcium phosphate poly (lactic-co-glycolic) acid (TCP-PLGA) scaffolds had homogeneous highly interconnected structure, with open macropores both horizontally and vertically. The average pore size of the macropores was about 450–500 μm and the porosity of the scaffold was about 70%. Figure 1 shows the SEM of TCP-PLGA scaffolds at the different magnifications.Some initial bone formation with partial filling of the defect area had to be reduced prior to scaffold insertion by a reciprocating saw. All scaffolds (n = 13) could be implanted and were fixed to the osteosynthesis plates by two screws each.

### Osteogenic differentiation of pig adipose derived mesenchymal stem cells (pADSCs) as monolayer and on scaffold

**Figure 2:**
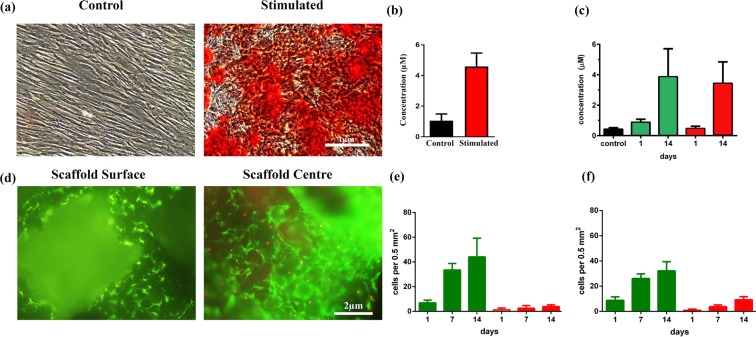
Alizarin Red and Live/Dead staining of pADSCs with quantification. pADSCs were cultured for 14 days in osteogenic differentiation medium in monolayer and on the scaffold. (**a**) Calcium deposition is shown as red colour by Alizarin Red staining of pADSCs cultured with or without osteogenic induction media (n = 3). Scale bar = 1 μm. (**b**) Quantification of the intensity of red colour in the monolayer (p < 0.0001). (**c**) Quantifi**c**ation of the intensity of red colour of Alizarin Red staining on the scaffold (surface vs. centre) at day 1 and day 14. Alizarin red staining reveals mineralized matrix both on the scaffold surface (green bars) and in the centre of the scaffolds (red bars, p = 0.53). (**d**) Cell viability by live/dead cell staining on TCP-PLGA scaffolds demonstrating the distribution of living (green) and dead (red) pADSCs on the surface vs. center. The green fluorescence represent living cells while red fluorescence indicate dead cells. (**e**) Quantification of ratio of living to dead cells on the scaffold surface where green represents the living cells and red represents the dead cells (p = 0.0225). (**f**) Quantification of ratio of living to dead cells in the scaffold center where green represents the living cells and red represents the dead cells (p < 0.0001). Scale bar = 2 μm. Data presented from 3 different pigs plated in triplicates.

After isolation and expansion, ADSCs clearly demonstrated their ability to differentiate into the osteogenic cell lineage. At day 14, alizarin red staining (ARS) confirmed osteogenic differentiation and matrix mineralization of ADSCs. Matrix mineralization was present in the cultures under osteogenic conditions while matrix mineralization was absent in the control medium. Cells grown as monolayer in osteogenic medium (OD) exhibited intense red staining while cells in the control media showed faint red colour **(**Fig. 2a**)**. Quantification of the ARS indicated that there was significant difference between the control and the osteogenic differentiation group where the osteogenic differentiation groups showed a 4 fold increase in mineralization compared to the control (p < 0.0001) (Fig. 2b).

Furthermore, osteogenic differentiation of ADSCs on the TCP-PLGA scaffold showed intense red staining indicating mineralized matrix both on the scaffold surface and the center compared to the control group. There was a tendency for higher alizarin red concentration on the scaffold surface than in the center both after one day and after 14 days as shown in Fig. 2c (3.86 ± 0.53 μM vs. 3.43 ± 0.41 μM, *p* = 0.53).

### Live/dead staining of seeded scaffolds

Live/dead staining was performed 14 days after seeding of the pADSCs on the scaffolds. The microscopic analysis of the fluorescent live/dead cells was shown in Fig. 2d, where living cells were seen as green fluorescence and dead cells were red. We have found far more living cells than dead cells both on central and peripheral areas of the scaffolds. However more living cells was detected on the scaffolds surface than at the center (44.08 ± 4.38 living cells vs. 32.17 ± 2.10 living cells, p = 0.0225) (Fig. 2e). At the center of the scaffolds, more dead cells were found than at the surface (9.25 ± 0.71 dead cells vs. 3.92 ± 0.40 dead cells, p < 0.0001) (Fig. 2f) expressed as number of cells per 0.5 mm².

### Clinical evaluation

One animal from the control group died prior to the first operation. After 12 weeks, two of the animals showed local signs of inflammation and therefore were excluded from further investigation. Consequently, 13 animals (control group; n = 6, test group; n = 7), which had a good general condition were finally included in the study and available for evaluation. All the pigs showed reduced activity and inadequate food intake on the day of the surgery. However, their activity and food intake returned to normal 1–3 days after the surgery. The animals were fed a pureed diet. All surgical wounds healed without swelling during the observation period. There was no postoperative infection or wound dehiscence. Gross examination revealed that the submandibular wound healed well. After dissection of the mandible, in the control group, the regions had bone margins that were easily identified from normal bone on observation and palpation. On the contrary, in the test group, the regions filled with tissue-engineered constructs could not be demarcated from adjacent native bone. The tissue filling of the defect was hard and non-compressible.

### Evaluation of new bone formation by microCT (µCT)

**Figure 3:**
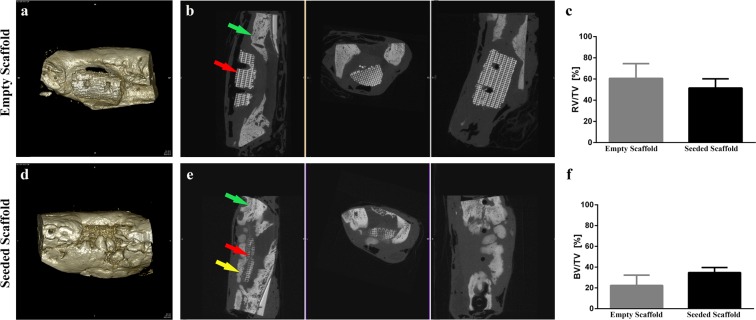
MicroCT (µCT) reconstructions and quantification of bone volume at 12 weeks after implantation in mandibular defects of minipig. (**a**) 3D reconstruction of the defect areas filled with the empty scaffold. (**b**) Transverse µCT of the defect area with the empty scaffold. (**c**) µCT quantification of the relative residual soft tissue volume to the total volume (RV/TV, p = 0.1766).). (**d**) 3D reconstruction of the defect areas filled with the pADSC-seeded scaffold. (**e**) Transverse µCT of the defect area with the pADSC-seeded scaffold. (**f**) µCT quantification of bone volume to the total volume of the former defect area (BV/TV, p < 0.05). Green arrows represent the bone of the mandible. Red arrows represent the scaffold implanted in the critical size mandibular defect. Yellow arrow represents de novo bone formation in the critical size mandibular defect.

The new formed bone in the former defect area was assessed by µCT and shown in Fig. 3. A significantly higher bone volume (BV) to the total volume (TV) of the former defect area was measured for the test group (n = 7) compared to the control group (n = 6) (bone volume to total volume, BV/TV, 34.8% ± 4.80% vs. 22.4% ± 9.85% respectively, *p* < 0.05).

The relative residual scaffold volume (SV/TV), corresponding to the amount of scaffold material that was not resorbed after 12 weeks, as well as the relative residual soft tissue volume (RV/TV) was higher in the control group but there was no significant difference on *p* = 0.05 level. (SV/TV, 16.98% ± 20.98% vs. 13.69% ± 8.83%, *p* = 0.7108 and RV/TV, 60.65% ± 13.92% vs. 51.50% ± 8.74%, *p* = 0.1766). The entire list of BV/TV of the 13 pigs were presented in Supplementary Table S1 online.

### Histological examination

**Figure 4:**
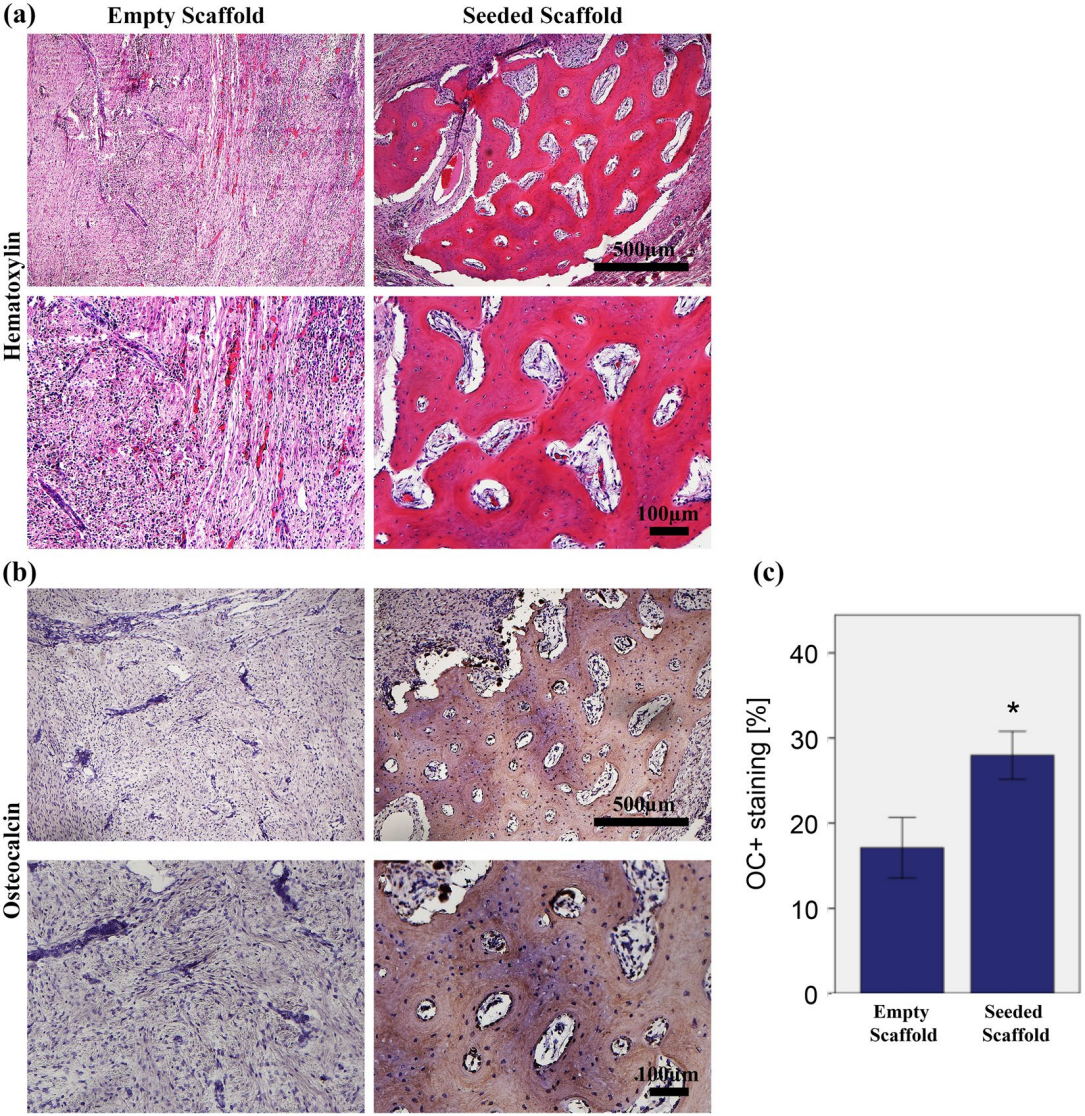
Bone regeneration capacity in the mandibular bone defect of minipigs evaluated by histological analysis and immunohistochemical staining at 12 weeks after implantation. (**a**) Haematoxylin and Eosin (H&E)-stained sections of the empty and seeded scaffolds at 5x and 10× magnifications at 12 weeks with scale bar = 500 µm and 100 µm respectively. H&E stain showed *in-vivo* new bone tissue formation with osteocytes. (**b**) Immunohistochemical staining for Osteocalcin in the empty and pADSC-seeded scaffolds at 5x and 10× magnifications (scale bar = 500 and 100 µm respectively). (**c**) Quantification of Osteocalcin staining. The area of bone labelling positive for OC was recorded in % of total bone area. Significantly higher amount of osteocalcin deposition was found in the test group (p < 0.001). Data presented as means ± SD (n = 3).

The qualitative evaluation of bone formation by Hematoxylin and Eosin (H&E) demonstrated proper integration of the newly formed bone into the host bone, also described as osseointegration in this study, as well as de novo osteogenesis in the scaffold centers in all of the seven ADSCs-seeded specimens. On the contrary, in the non-seeded control group, only 3/6 specimen demonstrated osseointegration and in 2/6 cases de novo bone formation was evident. The density of nuclei suggested that there might be an inflammatory reaction (Fig. 4a).

### Immunohistochemical assessment

The newly formed bone was assessed by osteocalcin immunostaining (Fig. 4b) in which quantification of the osteocalcin staining showed significantly higher amount of osteocalcin deposition in the test group in comparison to the control group (27.98 ± 2.81% vs 17.10 ± 3.57%, p < 0.001) (Fig. 4c).

## Discussion

Engineering the maxillofacial bones is challenging due to the presence of complex physiological structures such as sensory organs, facial skeletal features, cartilage and blood vessels. Moreover, clinicians have to control bacterial contamination in highly susceptible areas, including the oral and nasal regions^16^.

The regeneration of facial skeletal tissues must consider ways to ensure the restoration of aesthetics. Additionally, reconstruction should give sufficient mechanical strength and support movement due to speech and masticatory functions^16^.

Conventional means of repairing bone defects in the craniofacial region such as bone grafts, rigid fixation, and microvascular free tissue transfer for larger defects proved to be effective in small defects. However, those methods have significant morbidities and are not always successful for larger reconstructive problems^17^.

Recently, there are many exciting prospects that lie ahead for the reconstruction of craniofacial deficiencies including periodontal, alveolar ridge and large mandibular/maxillary discontinuity defects^18^.

The development of research in the area of bone augmentation have contributed significantly to the establishment of tissue engineering as a viable treatment option in dentistry such as alveolar bone, soft tissue of the teeth and dental implants^18,19^.

In the first part of this project (*in vitro*), we have established a protocol for isolation and culture of pig ADSCs, to assess their cell viability and differentiation capacity into the osteogenic lineage in 2D and 3D cultures. In the second part (*in vivo*), the potential of cell-scaffold assembly (TCP-PLGA scaffolds seeded with ADSCs) for the regeneration of extensive mandibular bone defects in a minipig model was assessed.

Critical size bone defects were created in the mandible of minipigs and, based on CT imaging, individualized CAD/CAM-fabricated TCP-PLGA scaffolds, fixed to the remaining mandible by titanium osteosynthesis plates, were successfully integrated in the defect area.

After 12 weeks, ADSCs-seeded scaffolds demonstrated significantly higher levels of bone volume than the scaffolds implanted without cell seeding (micro-CT analysis, osteocalcin staining). However, even in the ADSCs seeding group, only about one-third of the defects were filled with newly formed bone.

These results are of interest because bone tissue engineering (BTE) is a widely studied field and numerous approaches have been described that pursue the goal of successful bone regeneration. Suitable biomaterials have the function to provide the specific environment and matrix for bone formation and are based on a scaffold, respectively a combination of scaffolds, cells and/or drugs like growth factors^11^. Multiple factors such as biological characteristics, scaffold architecture, chemical composition and way of manufacturing influence the osteogenic capacity of scaffolds^11^. The scaffold used in our study is a synthetic biodegradable composite or hybrid, based on the ceramic tri-calcium phosphate (TCP) that is infiltrated with the polymer poly(D,L-lactide-co-glycolide (PLGA), abbreviated as TCP-PLGA. The infiltration of polymers such as PLGA significantly improves the mechanical properties in comparison to non-infiltrated TCP scaffolds, therefore balancing the problem of brittleness^20–22^. PLGA was chosen among other polymers because in a previous work of our study group it demonstrated favourable mechanical and biological behaviour^20^. The scaffolds were produced by 3D printing based on principles of CAD/CAM. That allowed for construction of high-resolution structures with interconnecting channels measuring about 450–500 μm. The porosity of the infiltrated scaffolds is 70–75%. Both the distribution and size of interconnecting channels as well as the high degree of porosity meet the requirements for BTE applications, and are well known prerequisites for vascular and bony ingrowth, oxygenation and flow of nutrients^23^. Apart from the role of micro-architecture, utilizing the CAD/CAM technology, 3D printing facilitates reconstruction of the complex morphology of craniomaxillofacial structures, which is vital in order to adequately restore function and aesthetics^24^.

A method of providing osteoinductive capabilities to a significant degree is the biological augmentation of scaffolds through the colonization with cells. Mesenchymal stem cells (MSCs) have proven to be very suitable for bone regeneration^25^. Apart from bone marrow derived mesenchymal stem cells (BMMSCs), the importance of adipose tissue derived mesenchymal stem cells (ADSCs) for BTE applications has increased over the last years.

The use of ADSCs has several advantages compared to BMMSCs. The harvesting via liposuction is a minimal-invasive procedure, a huge amount of cells can be obtained and fat tissue is easily replenishable, which is practical in clinical routine^9,26^. Furthermore, ADSCs are easy to cultivate and have a higher proliferation capacity^9,27^. The clinical experiences and laboratory results of our experiments indicate that harvesting as well as ADSCs isolation and proliferation procedures were feasible.

ADSCs clearly demonstrated osteogenic capacity, proving their character as mesenchymal stem cells^9^. According to other reports, ADSCs formed large nodules under 2D osteogenic culture condition^28^. Mean passage time of approximately 5–6 days is in analogy with other studies and significantly lower than with BMMSCs^29–31^. The fact that a large number of ADSCs is attainable is of special interest for the repair of large defects, as it was one of the challenges in this study. Approximately 1 × 10^6^ cells per 1 cm³ scaffold volume were finally seeded resulting in total of 5.0 × 10^6^ to 8.0 × 10^6^ ADSCs adapted to the individual scaffold size. The use of a modified seeding technique resulted in a successful ADSCs loading of the scaffolds with a high seeding efficiency. Over a period of two weeks, there was evidence for both a significant increase of living cells as well as an increase of the osteogenic capacity on the TCP-PLGA scaffolds *in vitro*. These results indicate a proper ADSCs adherence to the scaffold, normal function and good osteogenic properties. Interestingly, *in vitro*, proliferation and osteogenic capacity was also prominent in the center of the scaffolds. This is especially important when using large size cell-seeded scaffolds. Additionally, it would be most informative to know about the actual *in vivo* viability of the ADSCs in the scaffold center. That area represents an environment with a maximum level of hypoxia, comparable to central areas of fracture healing, taking seeded cells to a significant stress level^32^. However, a variety of studies describes a predominantly positive effect of hypoxia on proliferation and differentiation of MSCs and in particular ADSCs^33^. In a previous work, our study group demonstrated in 2D and 3D cell culture (3D cultivation on TCP-polyhydroxybuturate composite scaffolds) that proliferation capacity of both porcine BMSCs and ADSCs was higher under hypoxic conditions (2% oxygen) in relation to normoxic conditions (21% oxygen)^34^. With regard to osteogenic differentiation, BMMSCs showed highly decreased differentiation capacity under hypoxic conditions while ADSCs had a tendency towards increased osteogenic capacity^34^. These results are in line with those of a previous study dealing with hypoxic preconditioning of BMMSCs^35^.

So far, several experimental small animal models were performed to investigate the regeneration potential of ADSCs together with various scaffolds. The majority of these small animal studies indicate that the combination of ADSCs with different carrier materials has a beneficial impact on bone healing^36–46^.

However, the transferability of those results to humans is limited and no prediction with regard to the regeneration of challenging human extensive bone defects is possible. In contrast to small animal models, the minipig model used in this study resembles human physiology, bone regeneration rates and human anatomy, especially with regard to the shape and the dimensions of the mandibular bone^47–49^. Thus, it is possible to create a large size defect simulating a human critical size defect of the mandible. There are sparse data dealing with large size bone defects in the literature, in particular with respect to the field of craniomaxillofacial surgery. Viteau *et al*. tested the use of different scaffold materials with and without seeded MSCs in a large ectopic sheep model with the result, that new bone formation was only detected when the biomaterial constructs contained MSCs^50^. The bone defects created in this study in principle represents a “critical size defect” (CSD). CSD is defined as “the smallest intraosseous wound in a particular bone and species of animal that will not heal spontaneously during the lifetime of the animal”^51,52^. Still, there are inconsistencies concerning the geometry and volume in different research groups. For example Ma *et al*. stated a 2 cm long periosteum-removed segmental mandibular defect as CSD^53^. For a 4-wall defect in the left anterior mandible a perforating defect of 5 cm³ was considered critical^54^ as well as 5 cm³ defects in the mandibular angle region of growing pigs without buccal periosteum^55^. The results of Ruehe *et al*. indicate that 2 to 3 wall defects between 2 and 10 cm³ with a mucoperiosteal coverage at the anterior alveolar crest are not necessarily critical^56^.

In our study, the osteogenic capacity was significantly improved in the ADSC-seeded TCP-PLGA scaffolds compared to the non-cell seeded scaffolds. In the present study, the advantage of ADSCs-seeded scaffolds compared to non-seeded ones was evident with regard to osteocalcin deposition and newly formed bone in the former defect area. In order to enhance their osteogenic properties, we started *in vitro* differentiation of pADSCs into the osteogenic lineage over a period of 7 days prior to scaffold seeding and further implantation in the defect area of the animals. Schubert *et al*. demonstrated superiority of differentiated porcine ADSCs compared to non-differentiated ADSCs with regard to osteocalcin deposition and bone neoformation^30^ and the use of differentiated ADSCs is supported by further studies^38,57^. Interestingly, differentiated porcine ADSCs also outperformed differentiated and non-differentiated BMMSCs concerning cellular engraftment and formation of new bone in a porcine animal model^30^.

Despite the proven osteogenic advantage of cell-seeded scaffold, one must admit that the overall formation of new bone of about one-third in relation to the defect area after a period of 12 weeks was quite sobering, though one can speculate about further bone formation after 12 weeks. However, from a clinical point of view, a regeneration of 80% or more within a certain time should be considered necessary for a BTE application to act as a working alternative to autologous bone transfer. Especially for large sized defects, this remains quite a challenge.

However, what is the reason for the insufficient formation of new bone or the other way round how may the osteogenic capacity be improved? To our understanding, the key to successful regeneration of large bone defects or “critical size defects” in large animal models or human beings is controlling and modeling the hypoxic conditions within a BTE construct. While thinking about strategies to face the challenge of hypoxia in BTE, one should keep in mind two key facts: oxygen diffusion into avascular tissue is restricted to 200 µm and angiogenesis is as slow as 100 µm to 1 mm per day^58–60^. Although the ADSCs used in this study have angiogenic properties and can resist hypoxia to a certain degree with maybe even enhanced proliferation and differentiation capacities, this is seemingly not enough to overcome hypoxia. Besides, the TCP-PLGA scaffold architecture with its interconnecting channels allows for vessel ingrowth, but the centers of the scaffold have a distance of approximately 5–6 mm to the scaffold surface, which means that it takes at least 5 days until angiogenesis reaches the most hypoxic areas. Therefore, two general approaches are possible, with either increasing the oxygen supply or reducing the need for it in BTE constructs. The challenge of hypoxia may be resolved via (a) enhancement of vessel ingrowth into the BTE matrix by the local administration of VEGF or by optimizing the scaffold design, (b) prefabrication of an intrinsic blood vessel network or (c) “cellular protection strategy” through influencing the hypoxia signaling pathways^33^. With regard to large BTE constructs like those used in this study or even larger ones, a combination of prevascularization and modelling the cellular response to hypoxia seems to be a promising strategy. Especially the relative new field of “bioprinting” may serve as a key technology for further advancements in the field of prevascularisation^61^.

Besides hypoxia related challenges, mechanical properties and the ability to fix BTE constructs in the defect area are crucial in the context of large bone defects. Although all TCP-PLGA scaffolds in this study were applicable to the defect area and could be fixed well via titanium screws, a resorbable osteosynthesis system based on biodegradable polymers such as PDLLA or PLGA may allow for a better connection to the TCP-PLGA construct^62^. Even though the mechanical properties of TCP are improved by the infiltration of a polymer, these scaffolds still have the drawback of brittleness and further improvements are desirable^20^.

The inflammatory process plays an important role in the healing of the bone critical size defects. It is present at the very early stage attracting precursors for tissue regeneration and reduces its activity gradually as the process progresses. The histological examination of the empty scaffolds showed increased density of the nuclei suggesting an inflammatory response. Inflammatory responses play a crucial role in bone regeneration. However, prolonged inflammation retards the bone healing process^63^.

It can be concluded that the seeding of ADSCs on composite ceramic/polymer scaffolds improves bone regeneration in large mandibular defects than the use of empty scaffolds. We demonstrated, within *in-vivo* testing that seeded scaffolds had significantly enhanced bone regeneration compared to empty scaffolds after 12 weeks of healing. However, a considerable limitation of this experimental animal study is the need of further improvement with regard to the osteogenic and neo-angiogenic capacity is necessary in order to transfer this concept into clinical use and therefore overcome the “Valley of Death”, which describes the discrepancy between the large amount of studies and innovations in the field of TE and the sparse or even lacking routine clinical application and actual commercialization^64^.

Another limitation is the lack of characterization of the tissue at the repair site, which could be improved by performing fluorescent cell monitoring to detect and evaluate the distribution and migration of the cells inside the constructs or performing *in-vivo* histomorphometry by calcein blue and tetracycline to stain the existing bone and the new formed bone.

## Methods

### Ethics statement

This study was conducted according to the German Animal Welfare Legislation and the European Animal Protection Law (86/609/EEC). The experimental protocols used for the pigs were approved by the local animal committee of District Government of Upper Bavaria, Munich, Germany (Approval No: 55.2–1–54–2532–3–13). All animal handling and experiments were in accordance with relevant guidelines and regulations. All animal handling and experiments were in compliance with the ARRIVE Guidelines for animal research reporting of *in vivo* experiments^65^

### Animals

A total of 16 mixed gender skeletally mature miniature pigs (Münchener Trollschweine; average weight 85.0 kg, 12–14 month) were included in this study. All the animal experiments were performed at the Clinic for Swine, Center for Clinical Veterinary (Ludwig-Maximilians-University; LMU, Munich, Germany).

Prior to further experiments, animals were quarantined and housed in cages for 5–10 days for acclimatization. All animals were held under a constant 12:12-hours light/darkness regimen, where the temperature (22 ± 1 °C) and relative humidity (40–50%) were kept constant. The pigs were fed twice a day with pelleted commercial food and maintained ad libitum with water. Efforts were made to minimize animal suffering and to reduce the number of animals used.

After the operations, the food in the food pulp was mixed with water to facilitate food intake. For environmental enrichment the pigs were housed in stables on plain floor and straw (n = 2 pigs per stable) and in each stable a piece of soft wood was supplied on a chain. Additionally the pigs received colored plastic balls. Postoperatively, the pigs were housed individually but with visual contact to each other. All animals were postoperatively monitored under veterinary supervision.

### Study design

**Figure 5:**
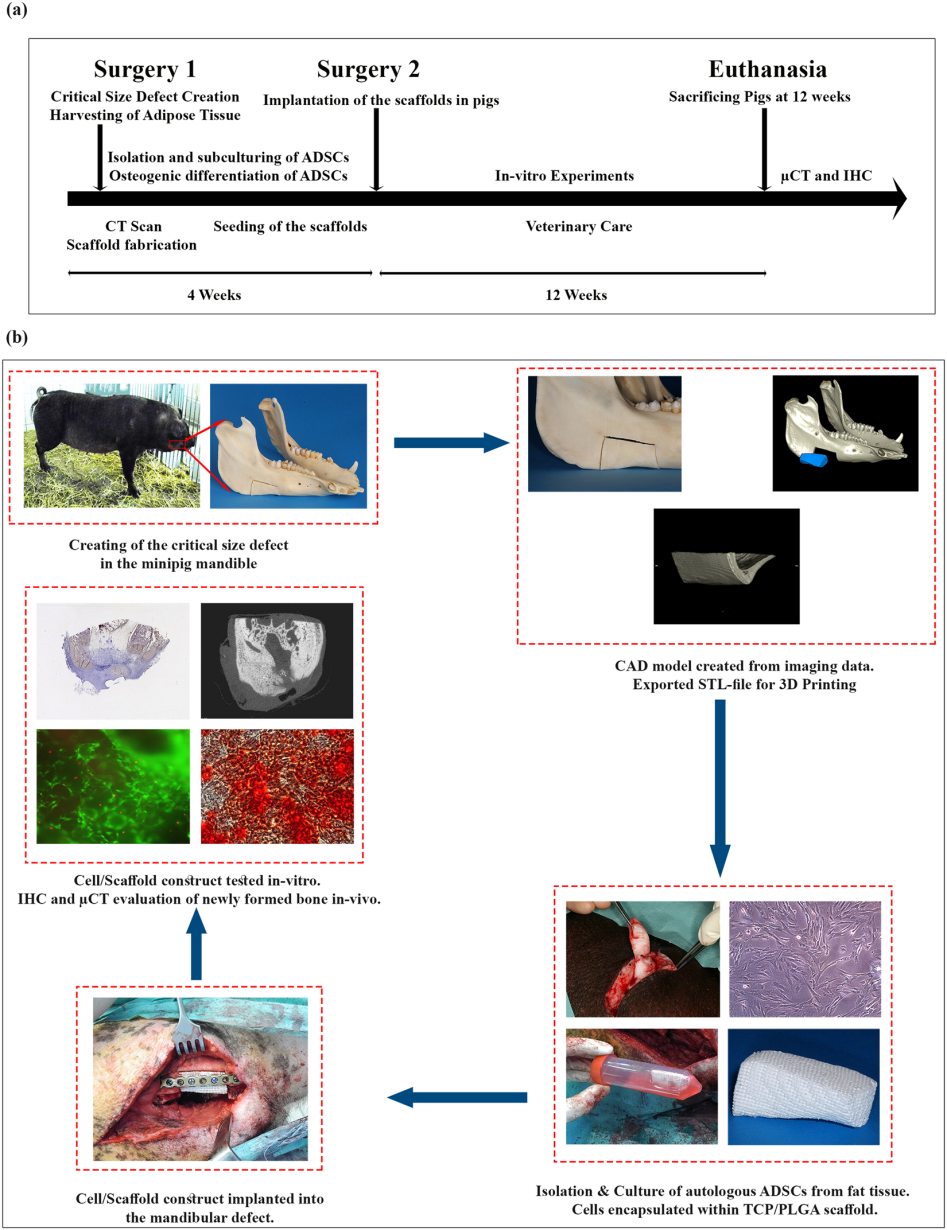
Timeline and graphical abstract of the experimental study. (**a**) Timeline and summary of the experiment. (**b**) Graphical abstract of the different working steps done in the experiment. Creating of the mandibular critical size defect, Fabrication of the scaffolds, isolation, cultivation and osteogenic differentiation of the cells, Implantation of cell-loaded scaffolds and healing, Radiographic, histological and immunohistochemical staining of the regenerated defects.

The study was performed in two phases. In the first phase, the mandibular critical size defect (CSD) was created and harvesting of the adipose tissue was performed. The second phase included scaffold seeding with cells and implantation in the critical size defect. Figure 5 shows the graphical abstract with the corresponding experimental timeline of the trial.

### Sample size calculation

An a priori power analysis was performed with G*Power Version 3.1.0 software^66^ and suggested a sample size of 8 animals per group for a power of 80% with a 5% level of significance.

### Mandibular defect model

The minipigs were randomly divided into two experimental groups:

1. Control group –empty scaffolds without seeded cells (n = 8/group).
2. Test group –scaffolds seeded with osteogenic differentiated pADSCs (n = 8/group)

### Surgical procedures

The surgery was performed under general anesthesia. To enable endotracheal intubation, the pigs received Propofol 1% (10 mg/1 ml MCT Fresenius, 3 mg/kg body weight, Fresenius Kabi Deutschland GmbH, Bad Homburg, Germany) via a peripheral venous catheter in the lateral ear vein. General anesthesia was maintained with inhaled isoflurane at the concentration of 1.5–2% (Isoba^®^ 1 ml/ml, Intervet, Unterschleissheim, Germany) after oral intubation. Atropine (Atropinsulfat B. Braun 0.5 mg/ml injection solution, 0.05 mg/kg body weight, B. Braun Melsungen AG, Hessen, Germany) was injected intravenously to avoid salivation and to stimulate cardiac action. Cardiopulmonary function was controlled during the operation by pulse oximeter. Furthermore, intraoperative analgesics (Metamizol; 40 mg/kg body weight, Vetalgin, Intervet Deutschland, GmbH, Unterschleissheim, Germany) was administrated. Following anaesthetic induction, prophylactic antibiotics was administered 1 hour preoperatively and for 2 days postoperatively to reduce the risk of infection (Streptomycin®, 0.5 g/day, Grunenthal, Stolberg, Germany). To maintain hydration, animals received a constant infusion of lactated Ringer’s solution while anaesthetized. Approximately thirty minutes prior to surgery, Azaperone (1 mg/kg body weight Stresnil, Janssen-Cilag GmbH, Neuss, Germany) were injected in the neck area of the pig intramuscularly as sedatives. Subsequently, Ketamin (10 mg/kg Ursotamin, Serum-Werk-Bernburg AG, Bernburg, Germany) was used as the intravenous anesthetic. For postoperative pain management, the pigs received Carprofen (Rimadyl® Pfizer Animal Health, 4 mg/kg body weight) orally for 3 consecutive days. All the monitoring of the vital signs and anesthesia were performed by experienced veterinarian (ME)

### Isolation and culture of porcine ADSCs (pADSCs)

After anaesthesia and asepsis, subcutaneous adipose tissue was harvested by surgical procedures from animals from the lower abdominal area of 12-weeks domestic minipigs, according to Yamamoto *et al*.^67^. The procedures were approved by the Ethics Committee of the Government of Upper Bavaria (55.2–1–54–2531.3–30–09).

Adipose tissue samples were immediately stored in sterile phosphate buffered saline (PBS, Invitrogen, Karlsruhe, Germany) supplemented with 5% penicillin/streptomycin (Invitrogen, Karlsruhe, Germany) and 2% amphotericin B (Invitrogen, Karlsruhe, Germany) and transferred on ice to the laboratory for further processing. The isolation, expansion and the differentiation of the ADSCs into the osteogenic lineage using differentiation medium was carried out *in-vitro* for 4 weeks before cell transplantation under good manufacturing practice (GMP) condition.

All isolation steps were performed within a laminar flow hood under sterile conditions. In summary, pADSCs were isolated from 20 g of adipose tissue by enzymatic digestion of fat. The harvested adipose tissue was cut into small fragments and subsequently mixed with 20 ml PBS containing 0.2% collagenase type II (Worthington, Lakewood, NJ, USA). Digestion was supported by gentle agitation on an orbital shaker for 2 hours at 37 °C. Meanwhile, a complete culture medium was prepared composed of Dulbecco’s Modified Eagle Medium DMEM- high glucose (DMEM-HG) supplemented with 15% fetal bovine serum (FBS, Sigma-Aldrich, St. Louis, MO, USA) and 1% penicillin/streptomycin (PAA Laboratories GmbH, Pasching, Austria).

Shortly after digestion, the cell suspension was filtered through a 100 µm cell strainer and an equal amount of complete culture medium was added to stop the reaction of collagenase. Then the cell suspension was centrifuged for 10 min at 1000 revolutions per minute (rpm) and the supernatant was removed and discarded. The cell pellet was resuspended in fresh complete culture medium. Anti-fungal agent (Patricin, 0.5 µg/ml) was added to the first passage to overcome fungal infection of the cultured cells. Four days later, non-adherent cells were removed by careful washing with phosphate-buffered saline (PBS) and the adherent primary cells were cultured for a further 7 days in complete culture medium. The cells were incubated at 37 °C in a 5% CO_2_ humidified. The culture medium was replaced every 3–4 days. When the cells reached 70–80% confluency, they were trypsinized and passaged until enough cells were obtained for the experiments.

### Mandibular critical size defect

**Figure 6:**
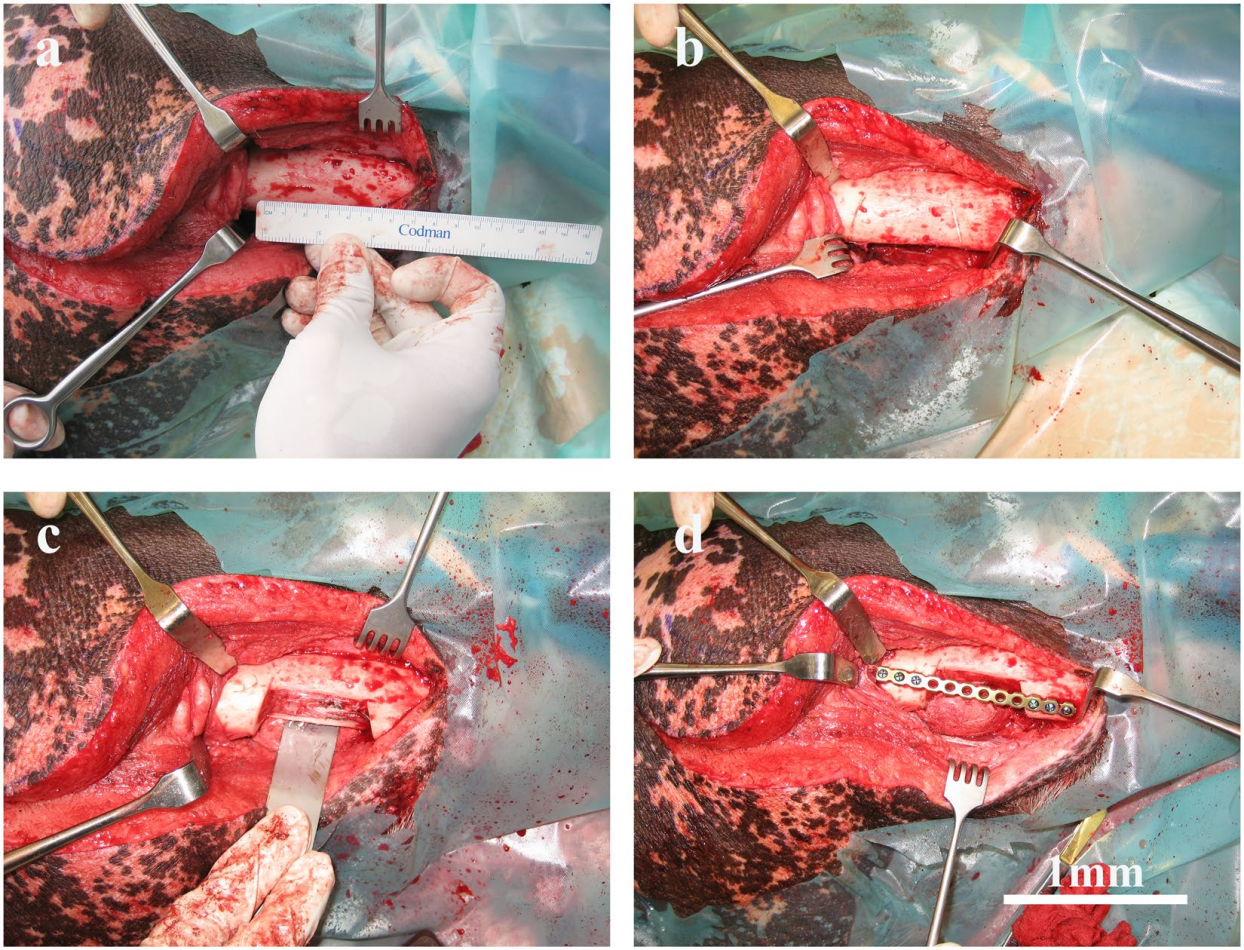
Surgical establishment of the mandibular critical size defect in the minipig model for implantation of empty and pADSCs-seeded TCP-PLGA scaffolds. An osteoperiosteal segmental mandibular defect of 1 cm length was made after adjustment of the titanium plate. (**a**) Submandibular skin incision with reflection of the mucoperiosteal flap exposing the body of the mandible with predetermining the dimension of the osseous defect using a ruler. (**b**) An initial cuts were done in the mandibular bone outlining the critical size defect. (**c**) Creating the mandibular critical size defect by cutting through the bone using reciprocating saw. (**d**) Fixation of the mandible with a load-bearing osteosynthesis plate to guarantee stability and avoid mandibular fractures. Scale bar = 1 mm.

A unilateral critical size defect was created in the pigs at the mandibular angle and posterior body region as shown in Fig. 6. These defects were approximately 6 cm^3^ in volume (anterior-posterior = 3 cm, buccal-lingual = 1 cm, inferior border-height of contour = 2 cm).

Briefly, the surgical site was scrubbed with surgical antiseptic and isolated with sterile drapes. A submandibular skin incision was made parallel to the inferior border of the mandible, followed by soft tissue dissection to expose the body of the mandible. The predetermined dimension of the osseous defect was measured and outlined using a ruler and a sterile marker. Using a reciprocating bone saw cooled with copious sterile saline, the mandibular osseous defects were created, followed by proximal periosteum dissection. The periosteum covering the defect site was completely removed. Proper surgical access to the mandible was provided with the help of cheek retractors. A load-bearing osteosynthesis plate (MatrixMANDIBLE™ Plating System, DePuy Synthes CMF, West Chester, USA) was applied at the lateral side of the mandible to guarantee stability and to prevent mandibular fractures. The lingual periosteum was preserved in all pigs. Finally, the incision was closed with simple interrupted sutures.

### CAD/CAM-fabrication of scaffolds

**Figure 7:**
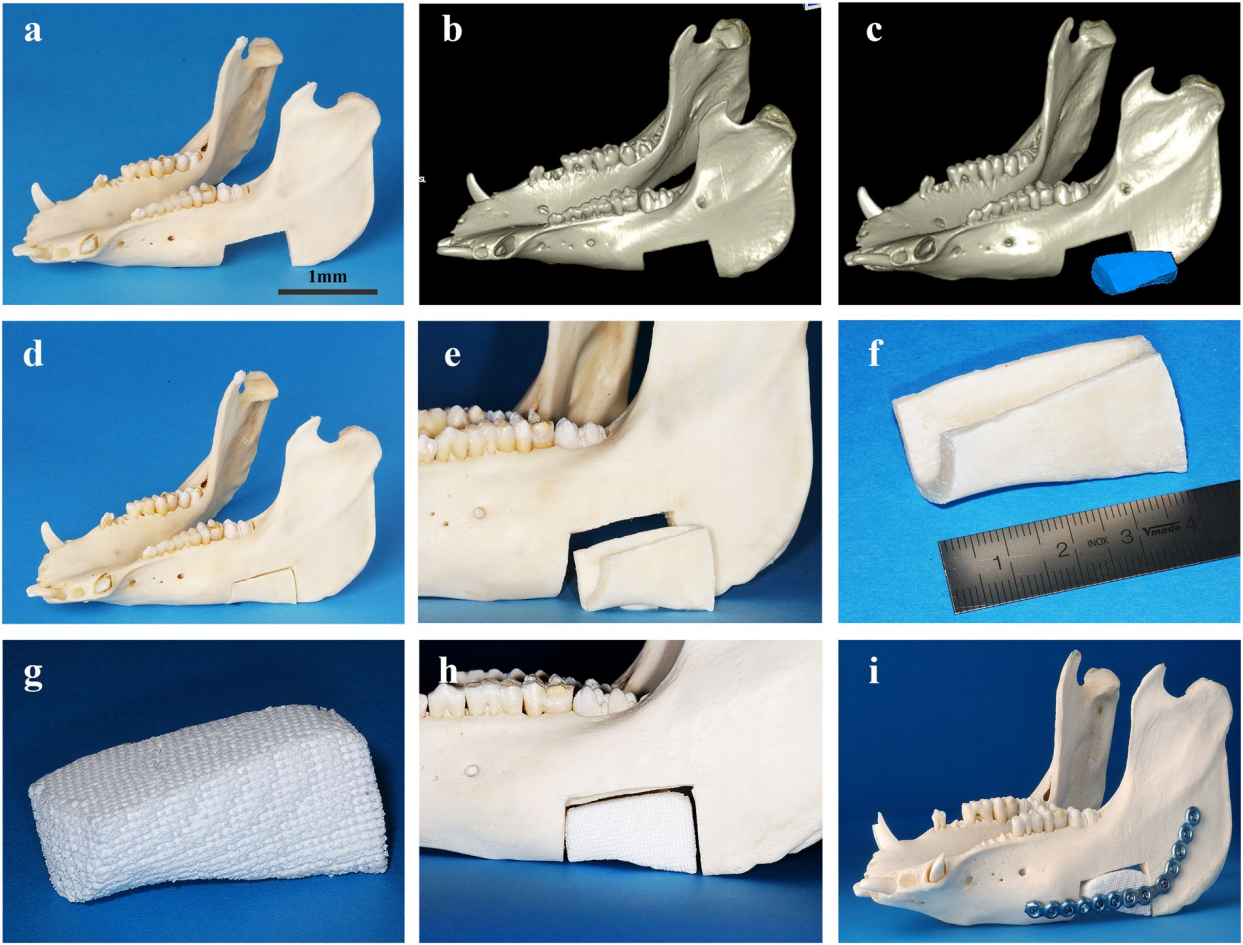
CAD/CAM workflow for the fabrication of the scaffold. (**a–f**) Exemplary depiction of a mandibular critical size defect, measuring about 6 cm³. Computer-aided design (CAD) of the scaffold and computer-aided manufacturing (CAM) of a corresponding scaffold fitted into the defect area. (**g–i**) The printed scaffold which is then fitted into the mandibular defect with fixation by a load-bearing osteosynthesis plate. Scale bar = 1 mm.

All 3D *in-vitro* experiments were carried out with TCP-PLGA cylinders of 20 mm height and 10 mm diameter (volume ~1.57 cm^3^) with interconnecting channels of 450–500 μm as previously described^20^. TCP-PLGA blocks were used in the *in-vivo* experiments. Figure 7 represent the schematic CAD/CAM manufacturing of the TCP-PLGA scaffolds.

Briefly, the removed bone segment was scanned by computed-tomography (CT). DICOM data were imported and further processed by means of the common 3D image processing application OsiriX (Pixmeo SARL, Bernex, Switzerland). Following segmentation of the CT datasets, a virtual scaffold model was created (computer-aided-design; CAD) and saved in STL format as it represents the standard format for rapid prototyping technologies. The STL data were transferred to the Chair of Fluid Technology and Microfluidics at the University of Rostock where customized TCP-PLGA (tri-calcium phosphate infiltrated with poly(D,L-lactide-co-glycolide)) composite scaffold were finally manufactured by CAD/CAM. Further scaffolds specifications were previously described^20^.

### Osteogenic differentiation of pADSCs as monolayer

Cells were counted and plated at density of 40,000 cells/well in six-well plates for osteogenic differentiation. After 24 hours, normal media were replaced with the osteogenic media for 14 days. The osteogenic differentiation medium was composed of Dulbecco’s Modified Eagle’s Medium- high glucose. (DMEM-HG) supplemented with 15% FBS, 1% penicillin/streptomycin (40IU/ml), dexamethasone (100 nM), ascorbic acid 2-phosphate (150 µM) and beta-glycerophosphate disodium (10 mM). As a control, pADSCs were cultured in complete culture medium without osteogenic reagents. The plates were incubated at 37 °C and 5%CO_2_ and the medium was changed every 3 days.

### Seeding of the scaffolds

Conditions for centrifugal seeding were modified from optimized methods described by Godbey *et al*.^68^. Prior to seeding, the scaffolds were submerged in 70% (v/v) ethanol under the laminar flow hood for 15 min. Following alcohol sterilization, scaffolds were washed three times in PBS.

In brief, the scaffolds were pre-wetted in culture medium for 4 hours at 37 °C in a humidified atmosphere of 5% CO_2_ in 50 ml Falcon tubes. The cells were resuspended in culture medium at the concentration of 5.0 × 10^6^ cells/scaffold. The tubes containing the pre-wet scaffolds received 20 ml of a suspension containing the desired concentration of cells by slowly pipetting of the correct volume of pADSCs suspension onto the top surface of each scaffold, covering the scaffold’s surface. The tubes were then loaded into the centrifuge and spun at 2500 rpm for 10 minutes. The spin was segmented into 5 times spinning, each is 2-minutes long. The spin was repeated to increase distribution of the cells on the scaffold. The cell-scaffold constructs were left in the 50 ml tube and gently placed in an upright position in the incubator overnight avoiding agitation of the tubes so that most cells adhered to the scaffolds.

### Osteogenic differentiation of scaffold-embedded pADSCs

The osteogenic differentiation of pADSCs was induced on the scaffolds for one day (d1) and two weeks (d14) with DMEM-HG supplemented with 15% FBS, 1% penicillin/streptomycin (40 IU/ml), dexamethasone (100 nM), ascorbic acid 2-phosphate (150 µM), and beta-glycerophosphate disodium (10 mM) all from Sigma Aldrich (Munich, Germany). Cells seeded on scaffold without osteogenic induction cultured for 1 and 14 days in complete culture medium without osteogenic reagents served as the control.

### Mineralization assay by alizarin red staining

Alizarin red staining (ARS) and quantification was performed per the manufacturer’s instructions with the osteogenic quantitation kit (ECM815, Merck Millipore, Darmstadt, Germany) to evaluate extracellular matrix (ECM) mineralization and the presence of calcium deposits in both control and osteogenic media. Briefly, the culture media were discarded, cells or scaffolds were washed with phosphate buffered saline (PBS) and fixed in 4% paraformaldehyde (PFA) (Sigma-Aldrich, Munich, Germany) for 15 min. PFA was then discarded and the cells or the scaffolds were washed three times with PBS and stained with the alizarin red staining and incubated for 20 min at room temperature (RT) with gentle shaking. Cells in the monolayer or the scaffolds were then rinsed with PBS to reduce non-specific staining and examined for mineralization nodules. Mineralized nodules were visualized and photographed with Axiovert 40 CFL microscope (Zeiss, Oberkochen, Germany).

### Quantification of alizarin red staining

For quantification of ARS in the monolayer and on the scaffolds, the osteogenic quantification kit (ECM815, Merck Millipore, Hessen, Germany) was used according to the manufacturer´s instructions. Briefly, 1.2 ml of 10% (v/v) acetic acid was added to the cells in the well plates or the cellular–scaffold construct and incubated at room temperature for 30 min with shaking. The lysate were transferred to a 1.5-ml microcentrifuge tube. After vortexing for 30 sec, the slurry was heated at 85 °C for 10 min and transferred to ice for 5 min. The slurry was then centrifuged at 14.000 × *g* for 25 min, and 375 µl of the supernatant was removed to a new 1.5-ml microcentrifuge tube. Later, 150 µl of 10% (v/v) ammonium hydroxide was added to neutralize the acid. Aliquots of the supernatant (100 µl) were placed in triplicates in 96 well plates. The osteogenic differentiation was calculated versus standard curve and the absorbance was measured at 405 nm using Multiskan FC microplate reader plate reader (ThermoScientific, Massachusetts, USA). The experiments were performed in triplicates from three different pigs.

### Live/Dead staining of seeded scaffolds

Cell viability was assessed by staining the cells with Calcein-AM/EthD-III using the Live/Dead fluorescent Cell Staining Kit II (Promokine, PK-CA707–30002, PromoCell, Heidelberg, Germany) according to the manufacturer’s protocol. In brief, pADSCs at passage 4 were seeded at a density of 5.0 × 10^6^ cells on cylindric 20 mm × 10 mm scaffolds (height × diameter). The cells were allowed to attach on the scaffold for 24 h. The scaffolds were washed twice with PBS, and sufficient volume of Calcein-AM/EthD-III staining solution was added to cover the scaffold. The scaffolds were incubated for 30–45 minutes at 37 °C in a humidified atmosphere of 5% CO_2_ protected from light. The cylindric scaffolds were cut perpendicular to their long axis in the middle of the scaffold into 4 parts and pictures were taken from the periphery and the central part of the scaffold using AxioObserver Z1 fluorescent microscope (Zeiss, Oberkochen, Germany). The experiment was repeated three times.

Cell survival were analysed by ImageJ (Rasband, W.S., ImageJ, U. S. National Institutes of Health, Bethesda, Maryland, USA, https://imagej.nih.gov/ij/, 1997-2018). Cells were automatically counted and divided into living and dead cells. Cell viability was measured at the surface of the scaffolds as well as in their center after one day, one week (d7) and two weeks (d14). Percentage of viable cells was calculated using the formula:

$$Percentage\,of\,viable\,cells=\frac{number\,of\,viable\,cells}{total\,number\,of\,cells}\times 100$$

### Bone construct implantation

**Figure 8:**
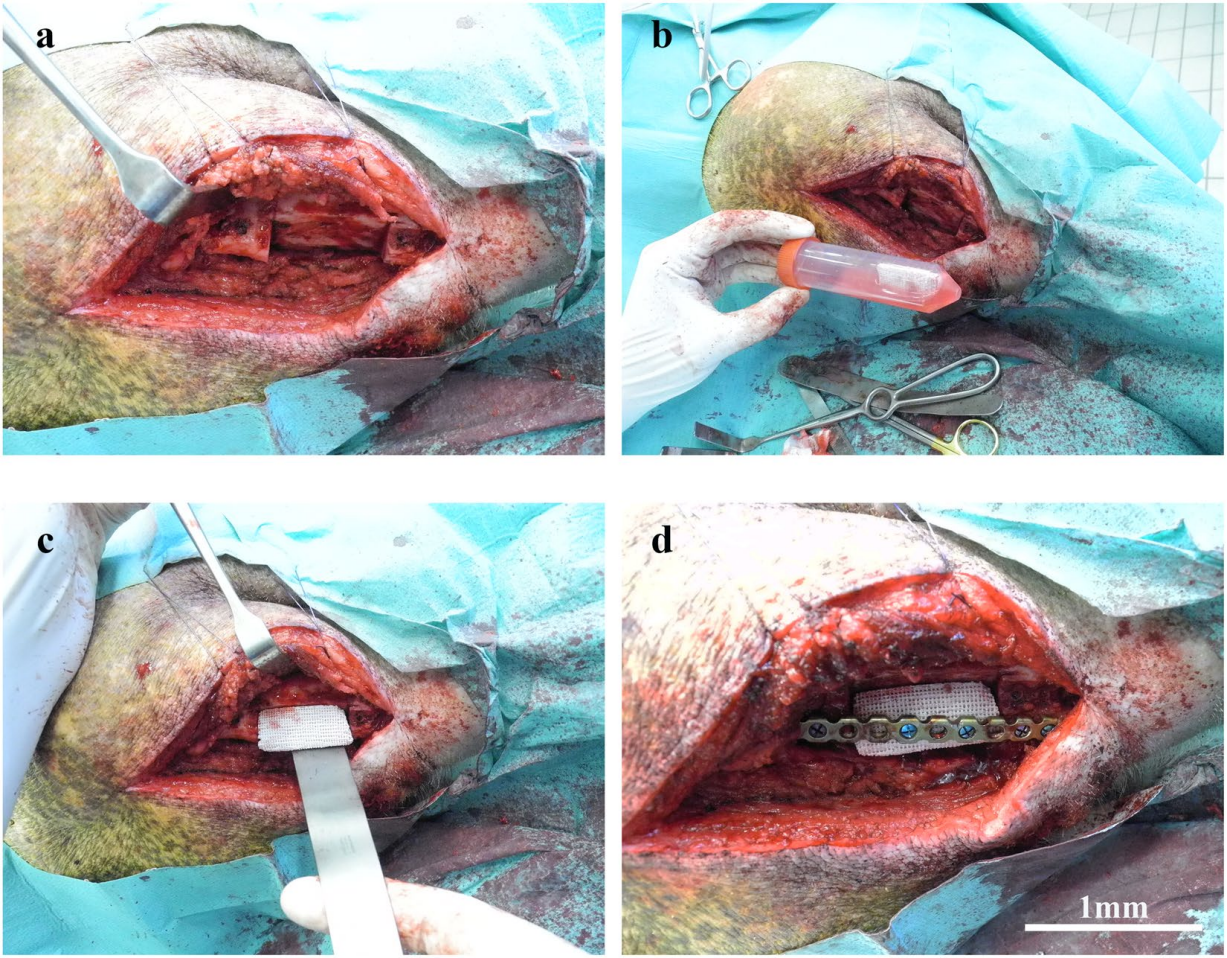
Implantation of the scaffolds in the critical size mandibular defects. The mandibular defects were reconstructed by implantation of empty and pADSC-seeded scaffolds in the minipig. (**a**) Reflection of the flap with exposure of the critical size defect. (**b**) The cell-scaffold construct being transported to the operating room in 50 ml falcon tubes with cell culture media. (**c**) Implantation of the scaffolds either empty scaffolds or cell seeded scaffolds in the critical size defect. (**d**) Fixation of the scaffold using a load-bearing osteosynthesis plate. Scale bars = 1 mm.

For the animal experiments, the cells were seeded on the TCP-PLGA blocks at a density of 5.0×10^6^ cells/scaffold. The cell/scaffolds constructs were then soaked in fresh complete culture medium, transported to the operating room and implanted under sterile conditions as shown in Fig. 8.

The same protocol for general anesthesia as well as for postoperative pain control and perioperative antibiotics was used as in the first operation. The animals were prepared and draped in a sterile fashion. The mandibular body and ramus were exposed through a submandibular incision. Constructs (cells/scaffold, n = 7) and control scaffolds (scaffold, no cells; n = 6) were implanted into each defect 4 weeks after the first operation. The scaffolds were attached to the load-bearing titanium osteosynthesis plates, which were previously placed at the lateral side of the mandible in the first operation. The scaffolds were friction-locked with two locking titanium screws. Closure was obtained in layers using 3-0 sutures. After the operations, the pigw were fed with food mixed with water to facilitate the food intake.

Twelve weeks after the implantation of the scaffolds, the minipigs were sacrificed in accordance with the ethical standards. The animals were injected with Azaperone (1 mg/kg body weight, Stresnil, Janssen-Cilag GmbH, Neuss, Germany) and Ketamin (10 mg/kg Ursotamin, Serum-Werk-Bernburg AG, Bernburg, Germany). Later, the animals were euthanized with Pentobarbital ((Release^®^ 300 mg/ml, injectable solution, WDT eG, Garbsen, Germany; 450 mg/5 kg body weight) by an intravenous injection via a peripheral venous catheter in the lateral ear vein. The mandible was harvested, examined and prepared for micro-CT (µCT) analysis and histologic evaluation.

### Micro-CT (µCT) analysis

The region of interest (ROI), defined as the former defect space with a surplus of at least 1 cm in all directions, was sawed out and specimen were fixed in 4% PFA for further evaluation of the implantation site by use of micro-CT (µCT), histology and immunohistochemistry.

To evaluate the formation of new bone, projection images of the fixed mandibles were obtained through a µCT scanner (µCT80, Scanco Medical, Bassersdorf, Switzerland) at with a resolution of 80 µm, a source voltage of 70 kV, and a current of 114 µA. Three-dimensional (3D) images were acquired and analyzed using OsiriX™ (Pixmeo SARL, Bernex, Switzerland, version 5.8.5, www.osirixviewer.com).

Qualitative evaluation of bone formation was carried out in addition to the quantitative assessment just described. Therefore, each data set was reviewed in coronal, axial and sagittal plane with regard to osseointegration as well as de novo bone formation in the defect center.

In order to define the volume of interest (VOI) for subsequent quantitative µCT analysis, the former defect area was matched to the postoperative µCT data sets. Then the defect regions were segmented and reconstructed three-dimensional with a spatial resolution of 80 µm per slice. Three different tissue types were distinguished in the µCT: bone, remaining scaffold material and soft tissue. A threshold and a bandwidth for every tissue type was determined according to clinically approved Hounsfield units (HU). HU up to 300 were considered soft tissue, HU between 300 and 700 were considered bone tissue and HU above 700 were considered scaffold material. After tissue segmentation, absolute volumes of each type of tissue/material were computed (bone volume; BV, residual scaffold volume; SV, residual soft tissue volume; RV and total volume; TV) as well as the percentage in relation to the corresponding total volumes.

Osseointegration was defined as proper integration of the newly formed bone into the host indicated by direct transition of new bone plus minus scaffold residuum and host bone. De novo bone formation was defined as island of newly formed bone within the scaffold center plus/minus bridging to peripheral formed bone.

### Histological examination

After µCT scanning, half of the mandibles were decalcified in 20% EDTA for 12 weeks, dehydrated in a graded series of ethanol (70–100%) and embedded in paraffin. Serial tissue sections with 8 µm thickness were prepared from the mid-sagittal plane of the defect area, treated with hematoxylin-eosin (H&E) and observed under a light microscope (Carl Zeiss Inc., Germany).

### Immunohistochemical (IHC) assessment

Apart from haematoxylin and eosin (H&E) staining, immunostaining was performed to evaluate the amount of bone formation in the defect area. The deparaffinized sections were blocked with 5% BSA and treated with Osteocalcin.

### Osteocalcin (OC) staining

The deparaffinized sections were washed with PBS, treated with osteocalcin (OC; 1:10000, mouse anti-pig osteocalcin monoclonal IgG, ab13418, Abcam, Cambridge, UK) and incubated over night at 4 °C (1:10000, mouse anti-pig osteocalcin monoclonal IgG, ab13418, Abcam, Cambridge, UK). The primary antibody was followed by incubation with the secondary antibody (1:200, horse anti-mouse IgG, BA-2001, Vector Laboratories, Burlingame, USA) at room temperature for 1 hr. A final incubation was performed using the tertiary complex streptavidin peroxidase (Pierce™ High Sensitivity Streptavidin-HRP, Catalogue no: 21130, ThermoFisher, Germany) for an additional 30 minutes. The reaction was visualized using Cell & Tissue Staining Kit (HRP-DAB system, R & D Systems, McCinley, Canada) according to manufacturer’s recommendations.

### Histomorphometric analysis

Relative osteocalcin staining was assessed within the region of newly formed bone adapted to a protocol by Sawyer *et al*. 2009^69^. A fixed threshold was applied in order to select positive staining within five randomly chosen regions of interest per specimen under Axiovert 40 CFL microscope (Zeiss, Oberkochen, Germany) at 10× magnification. Positive OC staining was set in relation to the total bone area of the selected region of interest.

### Statistical analysis

Statistical analysis was performed with SPSS version 21.0 (SPSS Inc., Chicago, USA). Results are expressed as mean ± standard deviation (SD). Student’s t-test was used for comparing means from two independent sample groups. A confidence level of 95% was used (p < 0.05).

## Supplementary information

Supplementary Information.

## Data Availability

All data generated or analysed during this study are included in this published article.
