# Supplementary material for: Bone regeneration of minipig mandibular defect by adipose derived mesenchymal stem cells seeded tri-calcium phosphate- poly(D,L-lactide-co-glycolide) scaffolds
Source: Sci Rep. 2020 Feb 6;10:2062. doi: 10.1038/s41598-020-59038-8 (PMC7005305; doi:10.1038/s41598-020-59038-8)
Supplement: Supplementary file 1 — Supplementary Information. [file 41598_2020_59038_MOESM1_ESM.pdf]

Bone regeneration of minipig mandibular defect by adipose derived mesenchymal stem cells seeded tri-calcium phosphate-poly(D,L-lactide-co-glycolide) scaffolds.

Florian Andreas Probst[1,2], Riham Fliefel[1,2,3,*], Egon Burian[2,4] Monika Probst[4], Matthias Eddicks[5], Matthias Cornelsen[6], Christina Riedl[2], Hermann Seitz[6], Attila Aszódi[2], Matthias Schieker[2], and Sven Otto[1,2]

Supplementary Table S1. The entire list of BV/TV of the 13 pigs.

**Identified Number**	**Pig no**	**Groups**	**Bone Volume (BV)**	**Total Volume (TV)**	**BV/TV**
1	495	Control	0.854	3.500	**0.244**
2	500	Test	1.930	5.436	**0.355**
3	504	Test	1.500	4.201	**0.357**
4	498	Control	1.475	4.400	**0.336**
5	489	Control	0.422	5.939	**0.071**
6	496	Test	2.016	5.508	**0.366**
7	503	Test	2.299	7.073	**0.325**
8	490	Test	2.591	8.332	**0.311**
9	507	Control	2.578	8.799	**0.293**
10	485	Control	1.996	7.803	**0.256**
11	492	Test	3.584	8.220	**0.436**
12	497	Test	1.335	4.650	**0.287**
13	499	Control	0.826	5.741	**0.144**

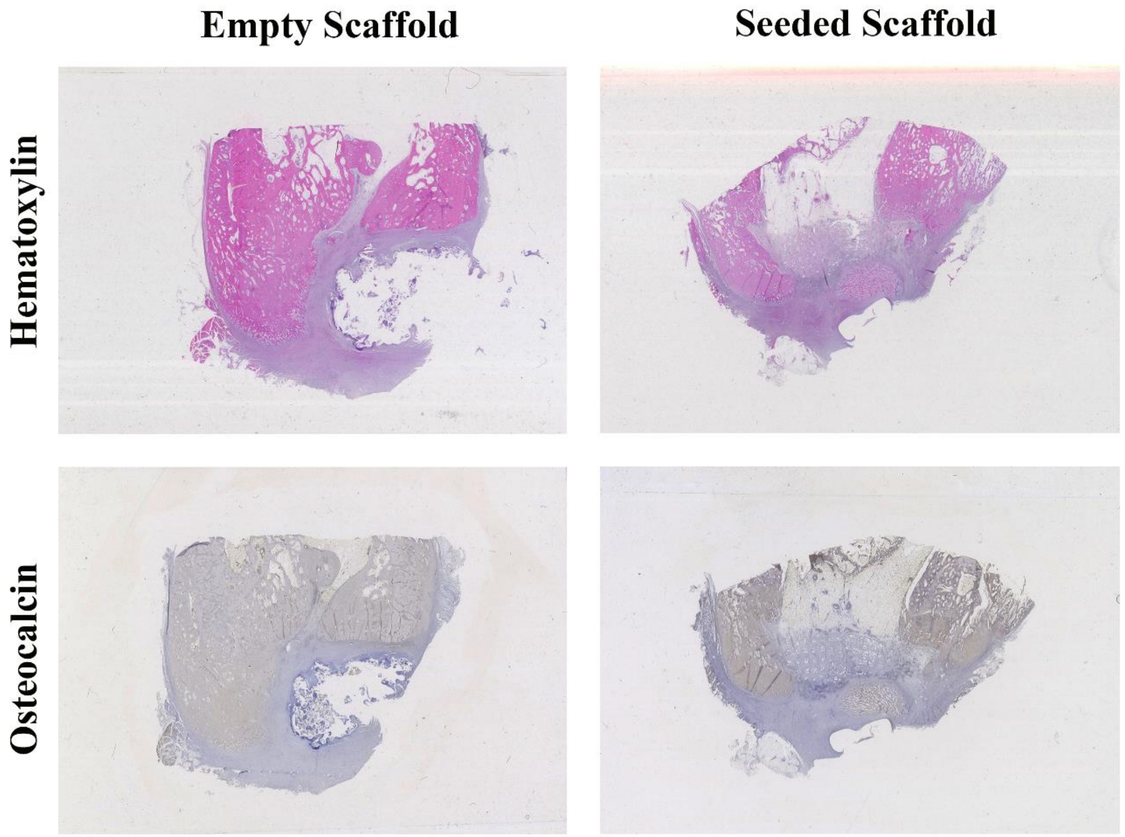

Supplementary Figure S1. Bone regeneration capacity in the mandibular bone defect of minipigs evaluated by histological analysis and immunohistochemical staining at 12 weeks after implantation. (**a**) Haematoxylin and Eosin (H&E)-stained sections of the empty and seeded scaffolds at 12 weeks. (**b**) Immunohistochemical staining for Osteocalcin in the empty and pADSC-seeded scaffolds. Data presented as means ± SD (n = 3).
